# Genomic and Epigenomic Instability, Fragile Sites, Schizophrenia and Autism

**DOI:** 10.2174/138920210793176001

**Published:** 2010-09

**Authors:** Cassandra L. Smith, Andrew Bolton, Giang Nguyen

**Affiliations:** Molecular Biotechnology Research Laboratory, Departments of Biomedical Engineering, Biology and Pharmacology, Boston University, Boston, MA, USA

**Keywords:** Genomic and epigenomic instability, fragile sites, schizophrenia, autism, folate, methionine, transulfuration, s-adenosyl methionine, cancer.

## Abstract

Increasing evidence links genomic and epigenomic instability, including multiple fragile sites regions to neuropsychiatric diseases including schizophrenia and autism. Cancer is the only other disease associated with multiple fragile site regions, and genome and epigenomic instability is a characteristic of cancer. Research on cancer is far more advanced than research on neuropsychiatric disease; hence, insight into neuropsychiatric disease may be derived from cancer research results. Towards this end, this article will review the evidence linking schizophrenia and other neuropsychiatric diseases (especially autism) to genomic and epigenomic instability, and fragile sites. The results of studies on genetic, epigenetic and environmental components of schizophrenia and autism point to the importance of the folate-methionine-transulfuration metabolic hub that is diseases also perturbed in cancer. The idea that the folate-methionine-transulfuration hub is important in neuropsychiatric is exciting because this hub present novel targets for drug development, suggests some drugs used in cancer may be useful in neuropsychiatric disease, and raises the possibility that nutrition interventions may influence the severity, presentation, or dynamics of disease.

## INTRODUCTION

Genomic instability refers to an increased mutation rate that can take the form of chromosomal abnormalities, translocations, large or small insertions or deletions and base changes. Epigenomic instability refers to perturbed responses of gene regulation to environmental fluctuations. Fragile site regions of the genome have high levels of genetic and epigenetic instability.

In 2003, we reported a link between somatic mutations (genomic instability) and fragile sites and schizophrenia [1]. Later, we reported aberrant epigenetic regulation of genes involved in dopamine metabolism in the synaptic cleft in schizophrenia and bipolar disease brains [2, 3]. Today, there is increasing evidence for genome instability in neuropsychiatric diseases, including an association with fragile site regions. Cancer is the only other disease associated with multiple fragile site regions, and genome instability is a characteristic of cancer. This article will review the evidence linking schizophrenia and other neuropsychiatric diseases (especially autism) to genomic and epigenomic instability and fragile sites. 

### Schizophrenia and Autism

Schizophrenia and Autism are neuropsychiatric diseases linked to multiple genetic and environmental factors. Like many common illnesses these diseases remain an enigma because there is no single factor or small number of factors that accounts for a large number of patients. 

**Fig. (1):**
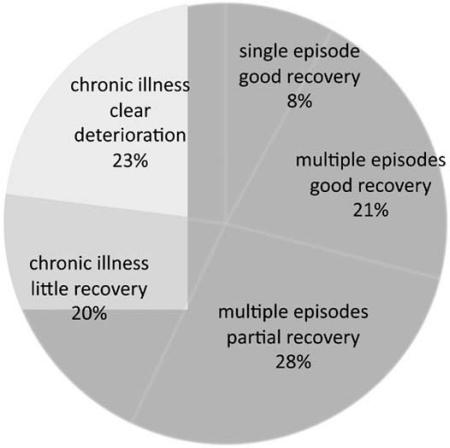
**Classification of schizophrenia based on outcome.** The outcome of schizophrenia disease is highly variable; suggesting different diseases may have been grouped together. (Adapted from Summary report of symposium “Schizophrenia and other Psychosis (http://www.science.org.au).

The prevalence of schizophrenia is ~1% worldwide but varies between 0.3 to 2.7% [4]. Diagnosis is based on the appearance and duration of about 30 symptoms divided into positive (e. g. hallucinations (especially auditory are common)), negative (e.g. withdrawal, blunted affect etc), and cognitive (executive function). However, symptoms (endophenotypes) and outcome (Fig. **1**) vary even in the same family, raising the possibility that several different diseases (i. e. the “schizophrenias”) presenting similar collections of symptoms have been grouped together [5, 6]. These and other observations suggest that a genetic predisposition is not sufficient by itself to cause disease. Further in some cases, the disease appears to be environmentally induced in the absence of detectable genetic predisposition (see below).

Autism is a complex, early onset (typically <5 years of age) lifelong illness that is difficult to diagnose and treat. Autism appears to be multiple diseases that make up autism spectrum disorder (ASD) defined by limits in three behaviors (1) social interactions, (2) communication and imaginative play, and (3) interests and activities. Other symptoms include impaired immunological responses, inflammation (especially in the gut), and oxidative stress [7]. Today, treatments include intensive educational and behavioral interventions with drugs to reduce remaining symptoms. 

## GENETICS

First-degree relatives of schizophrenia probands have a ~10% probability of becoming ill [8], while ~ 50% of cases of schizophrenia are spontaneous with no other affected family member [9]. Although variable [10-12], the general belief is that ~50% of monozygotic twins afflicted with schizophrenia are discordant for the disease, although progeny of both the well and ill discordant MZ twin have the elevated probably (~10%) typical of first degree relatives of ill individuals [13].

Genetic studies have linked many genes and chromosomal regions spread throughout the genome to schizophrenia in different families, but no single or small number of genes accounts for the majority of cases. Common alleles have small effects (e. g. ZNF804) while rare alleles (e. g. NRG1, DTNB1, DAOA and DISC1) have greater effects [14]. A summary of the genes linked to schizophrenia is shown in Table **1**. Genes linked to schizophrenia do not affect a single neurobiological system, and include neurotrophic factors (e. g. BDNF, NRG), neuromodulatory receptors (DRD, HTR), members of the synaptic packaging and release machinery (SNAP25), and both inhibitory and excitatory neurotransmitter systems (GRIN, GRIK, GABR). Also, there are genes linked to folate processing (MTHFR) and methylation (e.g. DNMT, COMT) see below. 

Except for mitochondrial defects in a subset of patients, no other common genetic or environmental factor, nor is an effective intervention linked to a majority of patients [15]. Clearly, there a genetic component with multiple genes linked to the disease (for reviews see [16] and [17]). Many genes linked to autism are similar to those linked to schizophrenia and bipolar disorder ([18, 19], http://neuropsych.bu. edu). 

## EPIGENETICS

**Fig. (2):**
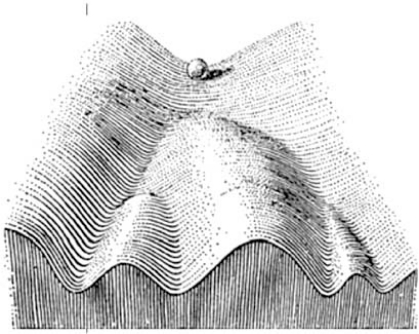
**Genetics, epigenetics, and development.** Waddington [20] coined the term epigenetics linking heritable factors to development. He likened development to a ball rolling down a valley, with epigenetic changes to DNA (DNA was proven to be the genetic material during this same period of time) directing a single genome towards different developmental outcomes, i.e. cell types. Epigenetic changes to DNA in a mature cell make development into another type of cell difficult (the ball cannot move into another valley).

Epigenetic programming refers to factors that are “epi”, or "on top of" genetic (DNA) sequences and was coined by Waddington in the 1940s to link genes and development [20] (Fig. **2**). Epigenetic regulation allows a single genome to code for functionally different cell types and short-term adaptation (for reviews see references [21-25]). In contrast, DNA sequence changes are responsible for long-term adaptation and evolution. 

The term “epigenetic programming” is evolving, and today refers to reversible molecular changes to DNA, RNA or proteins (e. g. histones) that regulate gene function but do not involve DNA base changes. Epigenetic changes include DNA methylation, RNA modification (e.g. editing (addition/deletion/change to base sequence), RNA interference) and both histone and non-histone proteins modifications (e. g. methylation, acetylation, phosphorylation, sumoylation, ubiquitination).

Epigenetic programming of chromatin begins shortly after DNA synthesis, although subsequent alterations may occur in response to variety of ordinary or pathological environmental or biological factors. Epigenetic changes occur globally early in development, and at specific loci throughout life and in disease states [26-28]. In cancer, the impact of epigenetic modification on gene expression has been studied for some time [29-35]. 

### DNA Methylation

**Fig. (3):**
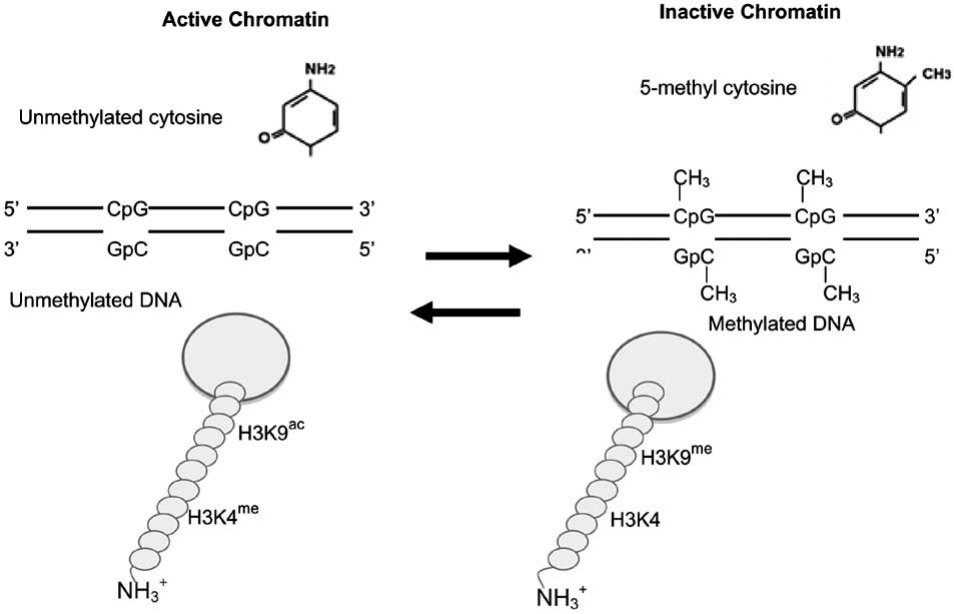
**Epigenetic programming to chromatin.** DNA methyl transferases (DNMTs) add methyl groups to the cytosines in CpG dinucleotide sequences. Histone 3 lysine 9 methylation (H3K9me) is concurrent with local DNA methylation in promoters. In the absence of promoter DNA methylation, histone 3 lysine 4 methylation (H3K4me) and histone 3 lysine 9 acetylation (H3K9ac) modification are found. Although both the DNA and histone modifications are reversible, only histone de-acetylases (HDAC) and de-methylases are known, no DNA de-methylase enzyme has been identified. Adapted from [25].

DNA methylation is the best-characterized epigenetic factor controlling gene expression (Fig. **3**; for reviews see [24, 25, 36-38]). In vertebrates, 4-8% of all cytosines, and 70% of cytosines within the 5'CpG3' dinucleotide sequence, are methylated. In contrast, 70% of the cytosines at 5'CpG3' dinucleotide sequences within promoter regions of active genes are unmethylated. There are ~29,000 "CpG islands" (regions rich in 5'CpGs3') in the human genome 2 sequence. The methylation state of half of these islands regulates mRNA expression. About half of these islands are highly methylated [39]. DNA methyltransferase (DNMT) enzymes are responsible for methylation of CpG sequences [40], with the rate of methylation determined by the availability of DNMTs and their relative affinity for a given CpG site on DNA [41], and other co-factors (see below). Today, no DNA demethylase has been identified. 

The number and location of methylated CpG sites in promoter regions usually, but not always, correlates with gene expression *in vivo* [24, 25, 36, 37, 38, 42]. Usually, dense DNA methylation is associated with irreversible silencing of gene expression, while a strong activator can overcome partial methylation. Partial promoter DNA methylation marks genes that may become unmethylated and expressed, allowing for re-adaptation to a changing micro- or macro- environment (e.g. season, ecological conditions, nutritional habits and demands of different developmental periods (see below)). More complexity in DNA methylation is introduced when the state of CpG sites within genes (i.e. outside the promoter regions) are compared to promoter dinucleotides. Ball *et al*. [39] show that methylation of CpG sites within genes is correlated with light promoter methylation; hence, gene body methylation appears to correlate with expression.

DNA methylation in promoter regions occurring at 5’CpG3’ dinucleotides within transcription factors recognition sites (e.g. GGG**CG**G and TGA**CG**TCA for factors stimulatory protein 1 (SP1) and cAMP response element protein (CREB), respectively) may decrease expression of genes driven by these factors [25]. Gene activation itself may impact local DNA methylation. For instance, transcription factor (e.g. SP1) binding may interfere with DNA promoter methylation directly [43]. 

Transcription can be *inhibited* by proteins that bind directly or indirectly to methylated DNA (see referenced reviews above). One methylated DNA binding family, consisting of the MeCP2, MBD1, MBD2, MBD3, and MBD4 proteins, has a conserved methyl-binding domain (MBD) and binds singly methylated CpG dinucleotides [44]. Another repressor family, all containing a zinc-finger motif, consists of Kaiso protein, which binds CGCGs, the Kaiso binding sequence (KBS; recognition sequence = TCCTGCNA) protein, and the ZBTB4 and ZBTB38 proteins that bind lone methylated CpGs dinucleotides [45].

Epigenetic changes in DNA are correlated with amino terminal histone 3 modifications (methylation and acetylation)(for reviews see [46, 47, 25]; Fig. **3**). Promoter regions of expressed genes (i.e., unmethylated regions) have histone 3 lysine-4 methylation (H3K4^me^) and histone 3 lysine-9 acetylation (H3K9^ac^) modifications. Promoter regions of unexpressed genes, (i.e. highly methylated regions) have no modification at histone 3 lysine 4 (H3K4) but have histone 4 lysine 9 methylation (H3K9^me^). 

Generally, chromatin codes (DNA and histone) are preserved through mitosis, although reprogramming may occur [48]. During meiosis and early development, complex differential global chromatin reprogramming occurs, some specific for male or female germline and others for development. Some germline epigenetic patterns are inherited [48].

Epigenetic programming imprints some genes to be expression in a parental origin dependent manner [47]. Gene imprinting is proven for ~80 genes, and predicted for ~200 genes (http://www.geneimprint.com). Most imprinted genes are associated with growth and development. In female cells, epigenetic changes turn off all gene expression from one X chromosome randomly in each cell during early embryogenesis [49]. This insures that chromosome X gene expression levels are similar for female (XX) and male (XY) cells. 

Although, epigenetic contributions to cancer phenotypes have been studied for some time, only recently has this area of research begun to impact neurological diseases. We and others have previously reviewed [24, 25, 50, 51] the connection between epigenetic modifications and neurological disease, including the effect of folic acid (a source of methyl groups for epigenetic modifications) metabolism on psychotic symptoms, and the co-morbidity of psychosis with diseases clearly linked to epigenetic changes (e. g. schizophrenia, bipolar disease, autism, Rett's and Angelmen's /Prader-Willi disease, mental retardation and degeneration (see below)).

## GENETIC AND EPIGENETIC REGULATION OF DOPAMINE METABOLISM

**Fig. (4):**
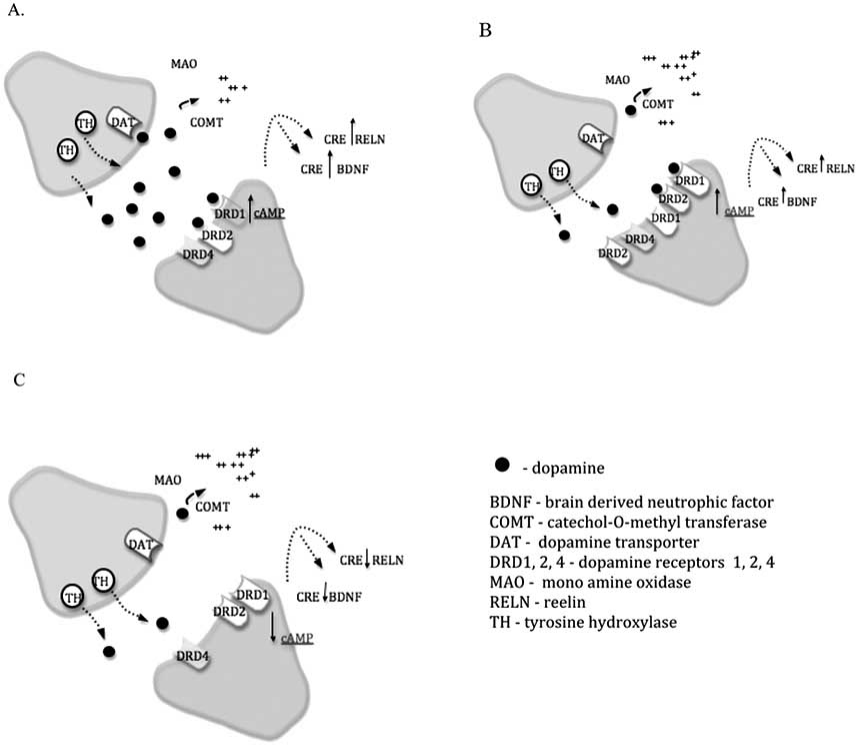
**Genetic and epigenetic regulation of dopamine metabolism in schizophrenia.** (**A**) Dopamine released by the pre-synaptic neuron into the synaptic cleft may dock with dopamine receptors on the post-synaptic neuron for downstream signaling; be degraded by MAO or COMT; or be taken back up into the pre-synaptic neuron by binding to DAT. (**B**) When dopamine degradation is high, for instance, by an increase in COMT activity, dopamine receptors expression is elevated to compensate for low amounts of dopamine in the synaptic cleft. (**C**) In schizophrenia, the coordinated up-regulation of the dopamine receptors does not exist, or exists at a greatly reduced level.

The dopamine hypothesis of schizophrenia arose because many anti-psychotic medications used in the treatment of schizophrenia are dopamine receptor antagonists. Oxygen methylation of dopamine by Catechol-O-Methyl Transferase (COMT) appears to be the prominent means of dopamine catabolism after synaptic release in brain regions such as the prefrontal cortex (reviewed in [52]). The 5’ region of the COMT gene contains methylation sites that are actively regulated. Our experiments [2, 3] studied promoter methylation and gene expression levels in Brodmann Area 46 (DL-PFC) of normal versus neuropsychiatric (schizophrenia and bipolar) individuals (Fig. **4**). The results revealed a significant correlation between membrane-bound COMT (MB-COMT) promoter hypo-methylation (especially at SP1 binding sites) and over-expression of the MB-COMT gene product in schizophrenia and bipolar disorder. 

The same samples used above were genotyped for a common COMT allele (Val158Met single nucleotide polymorphism (SNP)). The results showed that schizophrenia samples were more likely to have a VAL allele, and less likely to be homozygous for the MET allele than controls. Bipolar patients were more likely to be homozygous for the VAL allele than controls. 

The Val158Met polymorphism is known to directly affect the thermostability of the MB-COMT protein. The Met alleles is thermolabile, causing COMT enzyme activity in Met homozygotes to drop to approximately 1/3 the level of Val homozygotes at physiological temperature [53]. COMT hyperactivity (from the Val allele) has been linked to poor working memory as well as disturbed executive function and attention [54-58]. Genetic epigenetic gene expression results showed that dopamine degradation in the synaptic cleft is increased in individuals with schizophrenia because of increased COMT activity or expression.

Additional studies examined the expression and regulation of other genes involved in dopamine metabolism. The results revealed that expression of the dopamine receptor 1 (DRD1) was inversely correlated with MB-COMT expression in all groups, although to a lower level in the patient groups. DRD2 showed the reverse pattern: hypo-methylation of the MB-COMT promoter was nearly always associated with hypo-methylation of the DRD2 promoter and higher DRD2 gene expression levels. However, schizophrenia and bipolar patients show a significantly less severe decrease in methylation of their DRD2 promoters in response to MB-COMT hypo-methylation. 

Also, the promoter methylation state of the RELN gene was significantly linked to Val158Met genotype. All schizophrenics and control subjects possessing a Val/Val genotype had a hyper-methylated RELN promoter and a decrease in RELN gene expression. This is consistent with results [59, 60] that hyper-methylation of the RELN promoter and subsequent low expression of the reelin gene in the frontal lobes is correlated with schizophrenia. 

The fact that control subjects more strongly downregulate DRD1 expression and upregulate DRD2 expression when they possess a hypo-methylated MB-COMT promoter suggests that a mechanism exists for regulation of synaptic dopamine at the transcriptional level. Coordinated regulation was absent or decreased in neuropsychiatric patients. More recent unpublished data has detected aberrant methylation of the DAT1 and DRD4 promoters, but not the NRG1, HTR2A or NOS1 promoters, in samples from schizophrenic brains versus control subjects. The results suggest that aberrant synaptic dopamine metabolism in the schizophrenia/bipolar brain through genetic or epigenetic causes may contribute to disease pathogenesis.

Other groups have also examined methylation deficits in schizophrenia. For example, the methyltransferase DNMT1is up-regulated in the inhibitory inter-neurons of schizophrenia patients (reviewed in [61]). DNMT1 up-regulation is suggested to induce hyper-methylation and down-regulation of RELN and the GABA synthesizing enzyme GAD67 in prefrontal inter-neurons of schizophrenia patients. Woo *et al*. [62] and Costa *et al*. [61] speculated that down-regulation of the NMDA receptor subunit NR2A in these neurons may stem from hyper-methylation after DNMT1 up-regulation. RELN controls the surface expression of two other NMDA receptor subunits (NR2B and NR1, [63]) suggesting a possible deficit in NMDA receptors in the inter-neurons of schizophrenics. This supports the “NMDA hypofunction theory of schizophrenia” developed from observations that NMDA receptor antagonists, PCP and ketamine, both induce schizophrenia-like symptoms. In addition, the SOX10 (sex-determining region Y-box containing gene 10) gene, an oligodendrocyte specific transciption factor with a large CpG promoter island, is hyper-methylated and down-regulated in the prefrontal cortex (BA10) of schizophrenia patients [64].

## GENOMIC INSTABILITY

**Fig. (5):**
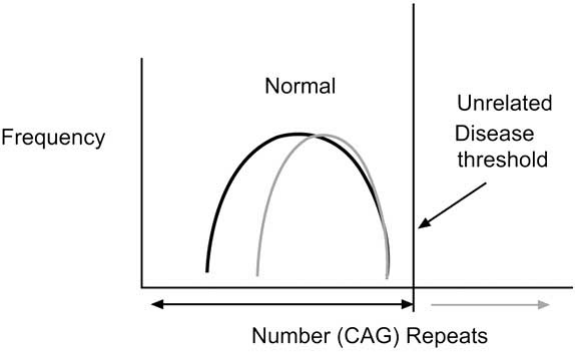
**Trinucleotide repeat distribution in individuals with schizophrenia.** Genes having (CAG)_n_ and (CCG)_n_ repeating sequences have been linked to specific diseases and to schizophrenia. The specific disease mutations are typical of repeat diseases where a repeat number over a threshold value (~50 repeats) leads to disease. Black = Distribution in unaffected individuals. Grey = In schizophrenia individuals, the repeat distribution is skewed towards larger sizes but not greater than the threshold value linked to specific disease.

Our initial research on schizophrenia focused on monozygotic twins. The goal was to understand disease discordance: how does one monozygotic twin avoid illness, and how do both the ill and well twin passed the same elevated genetic predisposition to progeny [1]. The specific aim was to identify, clone and sequence the expected small number of somatic changes present in monozygotic twins discordant for disease, and then do further studies to determine whether any differences were related to disease occurrence/presentation. The research targeted anonymous (CAG)_n _because these sequences are unstable and located within a number of genes linked to schizophrenia (e.g. [65-67], Fig. **5**). The experiments examined anonymous restriction length polymorphism (RFLPs) of PCR amplicons containing (CAG)_n _repeating and adjacent sequences in lymphocytes using a method developed by us called **T**argeted **G**enomic **D**ifferential **D**isplay (TGDD) [68]. TGDD is similar to differential display [69], but examines subsets of DNA sequences sharing a targeted sequence.

**Fig. (6):**
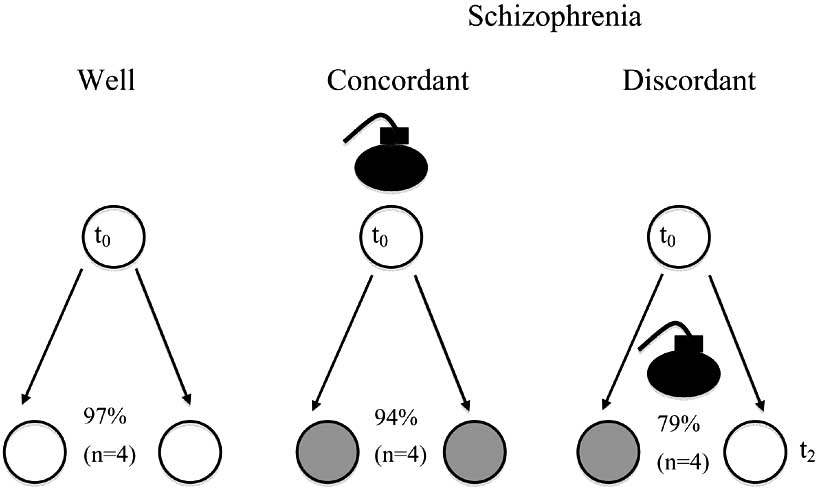
**Somatic genomic instability in twins affected by schizophrenia.** TGDD was used for RFLP analysis of genomic fragments containing (CAG)_n_ repeats and adjacent sequence in 12 pairs of monozygotic twins. The results showed that twins concordantly well or concordantly affected by schizophrenia had fewer differences than twins discordantly affected by schizophrenia. Assuming these twins began life as with identical DNA (i.e. are monozygotic), the observed differences represent somatic mutations, and the results show a higher somatic mutation rate in twins discordantly affected by schizophrenia.

Unexpectedly, a statistically significant high level of RFLP variability around (CAG)_n_ was detected in monozygotic twins discordant for schizophrenia (Fig. **6**). Twin pairs concordant for the disease had greater variability than controls, but for this small sample size this variability did not reach statistical significance. Assuming all the twin pairs were monozygotic (i. e., began life with identical DNA), RFLP variability must reflect somatic mutation rates after twinning. Hence, the results showed that a high somatic mutation rate was associated with schizophrenia, especially in monozygotic twins discordant for disease. 

Evidence supporting the idea include that schizophrenia is linked to genome instability. Cytogenetic observations of increased chromosome aneuploidy in brain cells from individuals with schizophrenia [70, 71] and other neurological diseases including autism, ataxia-telangiectasia [72, 73], Alzheimer's disease [72], Down syndrome, Edwards syndrome, Patau syndrome, Parkinson's disease, spinal muscular atrophy, mental retardation, Turner syndrome, psychiatric disorders associated with trisome X and Klinefleter syndrome, and 47,XYY karyotype (reviewed in [74-77]). Other evidence (reviewed in [1]) is the skewed (CAG)_n_ repeat distribution in schizophrenia (Fig. **5**), and the inverse correlation of disease with some cancer (reviewed in [78]).

More recently, genome wide scanning of SNPs in association studies revealed an elevated rate of copy number variation (CNV) in schizophrenia [79-82], and a number of other neuropsychiatric diseases such as autism, mental retardation, bipolar disease, Rett syndrome, Tourette’s syndrome, Prader-Willi/Angelman syndrome etc. (e.g. [18, 83], for review see [84]). Clearly, genomic instability is linked to neurological disease.

## FRAGILE SITES

**Fig. (7):**
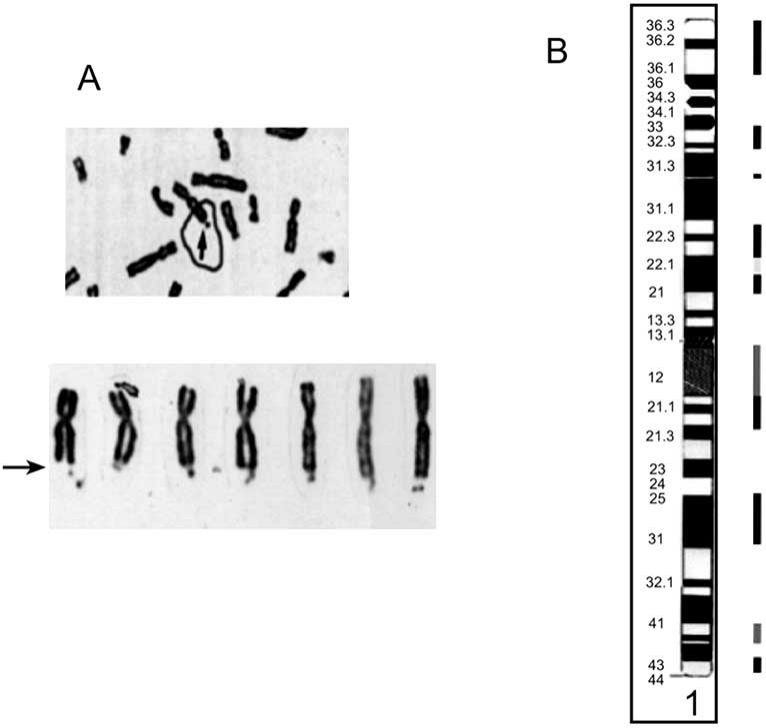
**Fragile site appearance and distribution. A**. Cytogenetic appearance of fragile X. Arrows point to fragile sites. **B**. Distributation of fragile sites along chromosome 1. The bars beside the cytogenetic bands represent the fragile site locations (see Table **1**). Dark to light bars represent inducing agents. Amphidicolin, 5-Azacytidine, and Folic acid, respectively. Taken from [150].

Fragile sites are regions of the genome that are prone to mutation and epigenetic changes; hence, hot spots for genomic instability. A fragile site is defined as unstable DNA stretch that appears as a gap or break on metaphase chromosomes (Fig. **7A**) when DNA replication of dividing cells is partially inhibited by incubation in culture medium deficient in folic acid or containing Bromodeoxyuridine (BrdU), distamycin, 5 azacytidine, or aphidicolin [85, 86]. 

Fragile sites are unusual chromosomal abnormalities because, although heritable, they appear only in a subset of cells, and usually only occur when induced. There are 119 known fragile sites (Tables **1** and **2**), spread throughout the genome classified as common or rare based on frequency in the population (greater or less than 5%, respectively). 

The first identified and best studied example of the association between fragile sites and mental illness is Fragile X syndrome. Fragile X syndrome is associated with transcriptional silencing of either FMR1 or FMR2 (Fragile X mental retardation genes 1 and 2) on chromosome X (for review see [87]). Silencing of FMR1 or FMR2 is accompanied by hyper-methylation of the (CGG)_n _expansion within fragile sites FRAXA at Xq27.3 or FRAXE at Xq28, respectively. The number and methylation status of the (CCG)_n _repeating sequences influences the expression of the fragile X mental retardation genes. The FRAXA and FRAXE promoter sites behave similarly. For FRAXA sites, well individuals have 7 to 50 (CCG)_n_ repeating sequences (with a mode of 30). Mental retardation occurs, and the fragile site becomes visible under folate deficient conditions, when the repeat number exceeds 230 and becomes hyper-methylated. Repeat numbers can reach up to 2000. Numbers between 50 and 200 are un-methylated and considered “pre-mutations”, but carriers may have symptoms other than mental retardation [88]. Schizophrenia is linked to several fragile sites (Table **4**), some of which are unique (e.g. [89]). Neurological diseases and cancers [90, 91] are linked to specific sites as well (Table **3**). 

Cells from schizophrenia patients grown in the absence of folate present a greater overall number of fragile sites per metaphase than controls [92, 93]. These results may indicate that schizophrenia patients may have a greater sensitivity to folic acid deficiency, or a higher number of fragile sites with borderline expansion (e. g. see (CAG)_n_ repeats in schizophrenia above). 

Most fragile sites are mapped only to the low-resolution chromosomal cytogenetic band level; ~15 fragile sites are characterized at the sequence level. One site appears to be ~3 million base pairs (bp) in size and contains 10 genes and multiple repeat sequences. Rare folate sensitive sites like FRAXA are composed of the expanded simple trinucleotide repeat (CCG)_n_ while some contain other interspersed repeats (e.g. LINE) or AT-rich sequences (e. g. common fragile sites are linked to AT-rich sequences). Replication of repeating sequences, or any sequence that deviates from the mean G+C level, can stress metabolism because the DNA replication machinery requires a different ratio of deoxynucleoside triphosphates (i. e. the ratio of G+C vs A+T). 

We calculated that ~70% of the human genome was devoid of fragile sites by determining what percent of the genome, at the cytogenetic band level, was linked to one or more fragile sites (Fig. **7B**). Our preliminary analysis [1] using chromosome abnormalities and genes linked to schizophrenia (reported in [94] and [95], respectively) found that ~70%, rather than the expected ~30% (X^2, p = 0.001), co-localize to regions of the having fragile sites. 

**Fig. (8):**
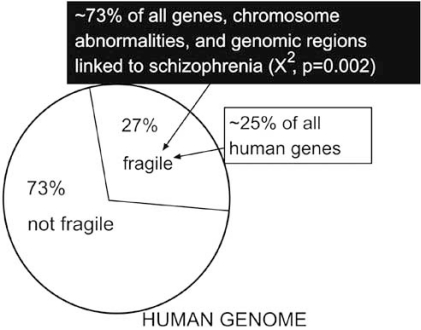
**Genomic distribution of genes, chromosomal regions, and chromosomal abnormalities linked to schizophrenia vs fragile sites.** These results were obtained by cataloguing genes linked to schizophrenia from a Pubmed search (http://www.ncbi.nlm.nih.gov) using the words "schizophrenia" AND "genes", "genetic studies", or "chromosomal abnormalities". The genomic regions that contain a fragile site was determined from a Pubmed search using the words "fragile sites". The genome "real-estate" of each locus and all the fragile sites was taken as the highest known chromosome banding resolution. Negative controls consisting of (a) all human genes and (b) genes tested but not found to be associated with schizophrenia did not have any preferential association with fragile sites.

More recent studies by us reviewed 387 genetic studies from the literature that identified 111 unique genes linked to schizophrenia (Fig. **8**). Of the 111 genes, 58 co-localized with at least one fragile site at the Giemsa band level (df = 1, χ^2^=14.227, p <0 .0001; Odds Ratio = 2.92). Moreover, a significant number of rare (CCG)_n _containing fragile sites co-localized with the sample of genes (df = 1, χ^2^=5.67, p < .025; Odds Ratio = 2.285). More detailed and updated information will be provided elsewhere. 

Expansion of repeating sequences within fragile sites is accompanied by local hyper-methylation (i.e. FRAXA and FRAXE) and the appearance of fragile sites *in vitro*. 

Certainly, genes in fragile sites regions in the brain may be impacted *in vivo *when individuals are folate malnourished during development. In adults, DNA replication in the brain occurs in the dentate gyrus and olfactory bulb, hence folate deprivation could impact neurogenesis during all periods of life, perhaps transiently increasing the severity of disease. 

In summary, fragile sites are more frequent in schizophrenia and co-localize with schizophrenia-linked genes. Fragile sites are sensitive to conditions that interfere with DNA replication, including folate deficiencies. Schizophrenia is linked to folate metabolism genetically (e. g. through hypoactive polymorphisms in genes that directly affect folate processing (e. g. MTHFR, MTR – see meta-analysis in [96])) and through epigenetic studies (see above) and environmental studies (see below). 

## ENVIRONMENTAL FACTORS AND SCHIZOPHRENIA

Some environmental factors linked to schizophrenia during early development are listed in Table **5**. No factor is sufficient by itself to induce disease. Family history, CNS damage, bereavement, and rubella infection increase the odds ratio most for disease. Paternal age and nutrition, well-documented factors linked to schizophrenia, provide important clues for understanding the biochemistry of schizophrenia. Further, the metabolic links can be used to postulate a role for other environmental components in disease (see below). 

### Paternal Age

**Fig. (9):**
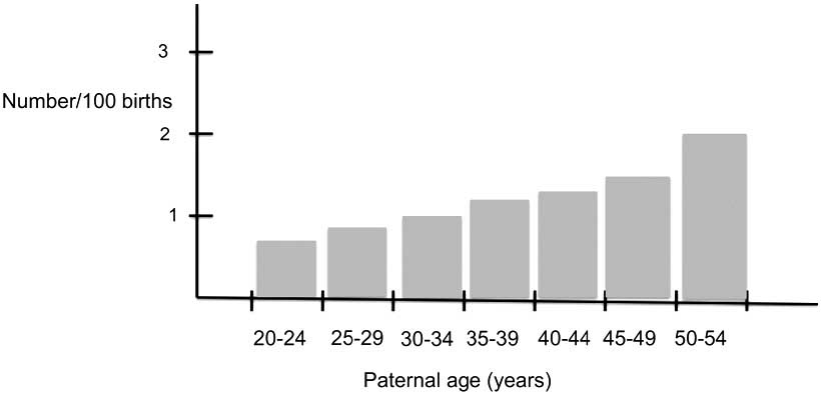
**The effect of paternal age on schizophrenia.** The data shows a linear increase in the incidence of schizophrenia and paternal age, and a three-fold increase for children of fathers over the age of 50. Figure is adapted from [97].

Since 1958, many studies have implicated paternal age as an environmental factor influencing the occurrence of schizophrenia (e. g. [97-99]). For instance, Malespina *et al*. [97] reported a three-fold increase in the incidence of schizophrenia in progeny of fathers over the age of 50 years (Fig. **9**). Today, the association with maternal age is unclear. Paternal and maternal age are linked to autism [100].

The paternal age connection implicates changes to paternal germline DNA in some cases of schizophrenia because DNA is the sole paternal biological contribution to progeny. Paternal aging is linked to diminished semen quality [101] and fertility [102], increases in sperm DNA damage (e.g. [103-105]) spontaneous abortions [105, 106], birth defects [106, 107] and singe base changes in rare autosomal dominant diseases [108-110]. For instance, mutations in DF1 fibroblast growth factor receptor (FGFR3) are linked to Achondroplasia. Mutations in FGF2 are linked to Apert, Crouzon, and Pfeiffer syndrome (PS), although some PS mutations may occur in FGFR2. Mutations in the lamina A (LMNA) gene are linked to Progeria, while mutations in REarranged during transfection (RET) are linked to multiple endocrine neoplasia (MEN2A MEN2B) and medullary thyroid carcinoma (MTC). 

Base substitutions account for all but progeria mutations in LMNA. The majority of mutations are transitions, (C to T) although some transversions (C to G) occur in a single dinucleotide CpG sequence. However, neither the number of replication cycles nor the observed mutation rates [110-113] accounts for the exponential rather than linear increase in disease as a function age; hence, it was suggested that these mutations confer a selection growth advantage to sperm. Lower and more linear-like increases as a function of paternal age are observed for a number of other rare autosomal dominant diseases such as neurobromatosis, bilateral retinoblastoma, Treacher Collins syndrome, multiple extostoses, and Sotos syndrome [108, 112, 113], as well as Down syndrome, neural tube defects, congenital cataracts, and reduction defects of the upper limb [105, 107].

### Nutrition

Under-nutrition (general caloric or protein deficiency) and malnutrition (deficiencies in specific elements, e. g. folic acid, zinc, copper, etc.) occur worldwide and are the most common diseases of childhood and prenatal life. Moderate to severe under-nutrition occurring prior to 2 years of age is associated with persistent behavioral and cognitive deficits that resist nutritional rehabilitation [114]. Pregnant mothers exposed to famine [115, 116] or malnourished (e.g. for folate deficiencies [117]) have an increased risk for children with schizophrenia. Maternal exposure to nutritional insults leads to persistent physiological and biochemical effects on the offspring [118-121]. Nutritional, factors that have been linked to schizophrenia and autism, like folate deficiency, can impact both genetics (DNA damage and fragile site expression) and epigenetics (DNA methylation *via *folate deficiency) in affected individuals. Generally, the specific mechanism(s) by which nutritional deficiencies produce these birth defects are unknown.

### Folic Acid

The importance of folic acid in preventing birth defects (e.g. neural tube defects including spina bifida) is well known, although the mechanism of disease induction is not understood [122]. Less well known is that fact that folic acid deficiencies are associated with a number of neurological diseases (e. g. [123, 124]) including schizophrenia and mood disorders [125-129], and are common in patients with psychopathology [130]. Furthermore, genes specifying proteins involved in folate metabolism are associated with schizophrenia and mood disorders as well as autism and other neuropsychiatric diseases [131]. Folic acid provides methyl groups to form S-adenosyl-methionine (SAM, see below), the universal intracellular methyl donor during methylation reactions such as those important in epigenetics.

### Folic Acid Metabolism

**Fig. (10):**
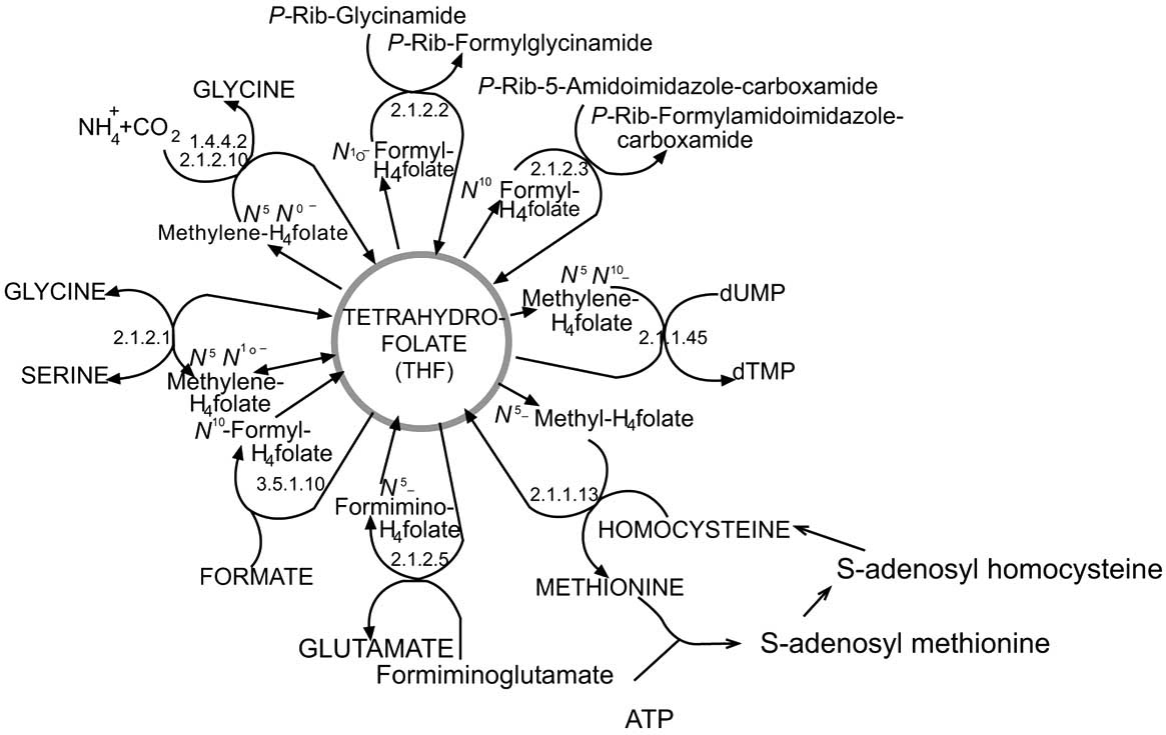
**Folic Acid Cycle.** Folate is an essential nutrient that is required in the synthesis of nucleic acid, s-adenosyl methionine (SAM) and amino acids. Further, synthesis of these monomers and their incorporation into polymeric molecules most times requires activated nucleosides like ATP, NAD and GTP whose synthesis depends on folic acid intermediates. Hence, the synthesis of DNA/RNA and SAM is heavily dependent on folic acid. (Figure adapted from http://www.tcd.ied/ IUBMB-Nicholson/pdf/29.pdf).

At the molecular level, folic acid deficiencies have the potential to disrupt nucleic acid metabolism, processes that require energy (i.e. ATP or NAD, GTP), activated nucleotide precursors (ribo - and deoxyribo- nucleoside triphosphates, e. g. DNA replication and RNA transcription), or SAM (or folate directly) for methylation (Fig. **10**). Abbreviated schemes of de novo synthetic pathways for ribo- and deoxyribo- nucleoside triphosphate synthesis are shown in Fig. (**11**). Folate derivatives are required by thymidine synthase that converts dUMP to dTMP, and for two steps in the purine biosynthetic pathway to make IMP; hence impacting ribo and deoxyribo purine synthesis. 

**Fig. (12):**
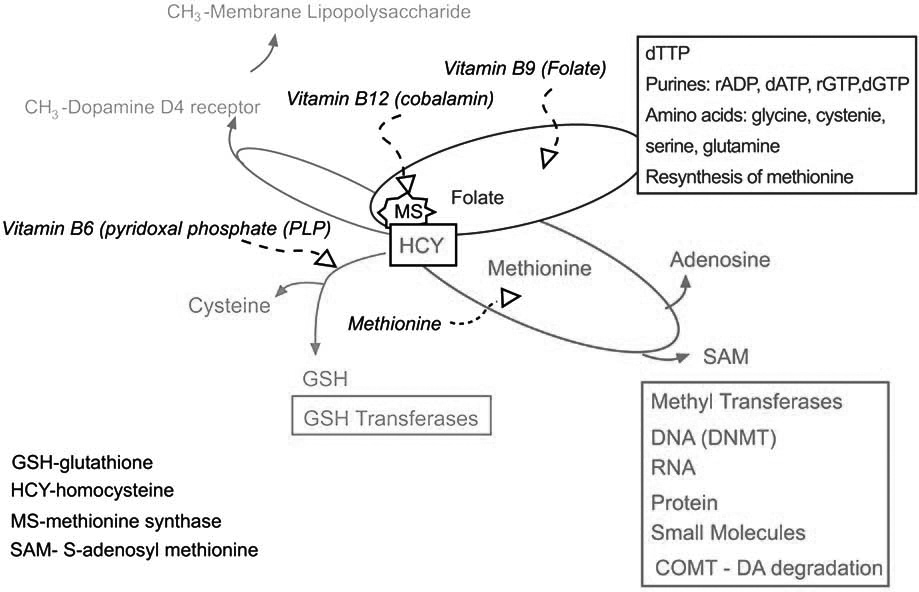
**Confluence of the folate, methionine, trans-sulfuration, and dopamine D4 receptor methylation pathways.** Folate is converted to derivatives that are utilized for the synthesis of dTMP, and IMP, and the amino acids serine, glycine, methionine and glutamate. SAM is formed from methionine and adenosine in the methionine cycle. Homocysteine (HCY), a degradation product of SAM, is converted to methionine by the enzyme methionine synthase (MS), utilizing a folate derivative, or by betaine homocysteine methyl transferase (BHMT) utilizing betaine (a choline derivative) as a methyl donor. In addition, HCY is a precursor for the biosynthesis of cysteine and the primary intracellular antioxidant, glutathione (GSH). The enzyme MS covalently adds a methyl group to the dopamine D4 receptor (DRD4), which transfers the methyl group to lipopolysaccharides. In mammals, folate, methionine, and vitamins B6, B9 and B12 required by these pathways, must be obtained from the diet or intestinal bacteria. Methionine may also be obtained from degradation of proteins.

Folate participates in the methioine cycle to synthesize S-adenosyl methionine (SAM). SAM is the second most used cofactor in the cells after ATP (Fig. **12**). SAM is used by >100 methyl transferases that act on DNA, RNA, proteins (e. g. DNA methyl transferase DNMT (for review see [40])), histone methyl transferases (HMT), and small molecules (e.g. COMT), and for the synthesis of polyamines that stabilize DNA. 

In the methionine cycle, a methyl group from folate is use by the enzyme, Methionine Synthase (MS), to convert homocysteine (HCY) to methionine. Alternatively, Betaine Homocysteine Methyl Transferase (BHMT) regenerates methionine from HCY using a methyl group from betaine (choline). Dietary and regenerated methionine reacts with ATP to generate SAM, while HCY is the product of de-methylated (*via *methyl transferases) and de-adenylated SAM.

Besides being used to reform methionine, HCY may be directed towards the trans-sulfuration pathway to produce the amino acid cysteine, and the primary intracellular antioxidant, glutathionine (GSH) HCY is up-regulated in schizophrenia patients with a 5 microM plasma HCY level associated with a ~1.7 fold increase in schizophrenia risk [95]. 

MS, the enzyme that uses folate to reform methionine from HCY, covalently adds a folate derived methyl group to the dopamine D4 receptor. The dopamine D4 receptor acts like a methyl transferase when activated by dopamine and transfers the methyl group to membrane lipid polysaccharide, changing local membrane fluidity [131]. Dopamine function and metabolism is therefore tied to the folatemethionine-transulfuration metabolic hub in multiple ways: directly, through dopamine degradation by COMT, and indirectly through dopamine D4 receptor methyl transferase activity and promoter methylation of genes active in dopamine metabolism in the synaptic cleft.  This metabolic hub (Fig. **12**) links DNA replication and epigenetic changes through folate and SAM metabolism, and because epigenetic marking closely follows DNA replication at the macromolecular level. HCY, a key intermediate used for SAM metabolism, is required for the synthesis of GSH; hence, dopamine metabolism, DNA replication and epigenetic marking are linked to oxidative stress.

The brain is especially sensitive to oxidative stress. Oxidative stress (hypoxia) is linked to schizophrenia directly (Fig. **9**), is a common consequence of obstetric complications linked to schizophrenia [132], and a potent inducer of fragile sites and genomic rearrangements [133]. Hence, oxidative stress through the transulfuration pathway is linked to DNA metabolism, and epigenetic marking. For instance, increased oxidative stress can direct HCY toward GSH production rather than SAM production, impacting many processes *in vivo*. 

Nutrition is critical for maintaining the folate-methionine-transulfuration hub because vitamines B6, B9 (folate) and B12, and the amino acid methionine must be obtained from the diet. Other factors listed in Fig. (**9**) can impact the folate-methionine-transulfuration hub. For instance, winter births are associated with times of food scarcity [134], and many times bereavement and depression are accompanied by reduced food intact. Infection or inflammation increases metabolites requirements such as those needed for DNA replication, or transcription.

Aberrant folate metabolism in schizophrenia has been demonstrated in a number of studies, for review see [135, 136, 2]. In fact, the Nobel Laureate (twice), chemist Linus Pauling, advocated for nutritional interventions in psychiatry in the 1960s [137]. 

Aberrant folate metabolism has been detected in autistic patients. In an impressive series of experiments, James *et al. *[138-141] detected aberrant levels of metabolic markers for the folate-methionine-transulfuration hub in patients and their mothers. For instance, decreased levels of methionine cycle (e.g. methionine, SAM, S-adenosylhomocysteine (SAH), adenosine, and HCY), and trans-sulfuration pathway (e. g. cystathionine, cysteine and total glutathione (oxidized (GSH) + reduced GSSG)), metabolites were detected. Also reported was an increase in other methionine cycle (e.g. SAM, adenosine) and transulfuration (e.g oxidized glutathione) pathway metabolites. In 2006, James *et al*. [138] linked SNPs in genes within the folate cycle (in the reduced folate carrer (RFC), methylenetetrahydrofolate reductase (MTHR), the methionine cycle (COMT), and the transsulfuration pathway (glutathionine-S-transferase (GST) to autism. In a preliminary study, James *et al*. [141] demonstrated that a nutritional treatment regime (supplementation with methylcobalamine (methylated vitamin B6), and folic acid) improved but did not normalize abnormal metabolite blood values. An analysis of the effect of nutritional supplementation on disease symptoms was not measured, although anecdotal improvements were reported.

## CONCLUSION

In summary, genetic and environmental components of schizophrenia and other neuropsychiatric diseases point to the importance of the folate-methionine-transulfuration pathway. This idea is exciting because this hub presents novel targets for drug development, and may lend themselves to nutrition interventions. 

Folate supplementation has been successful in the prevention of spina bifida and related abnormalities. Similar therapies may decrease risk and severity for neuropsychiatric disease. Faulty DNA replication and epigenetic marking during brain development and adult neurogenesis may impact occurrence, presentation and dynamics of neuropsychiatric disease. Simply providing excess folate may not be useful (see [142]). 

Reed and colleagues [143-146] have developed a dynamic model of the interaction of the folate and methionine cycles at the protein level. The Reed model is consistent with published data but does not yet include the entire folate-methionine-transulfuration hub, nor has the model been tested experimentally. However, this model is a beginning, and reminds us that an understanding the complex, dynamic behaviors of metabolic pathways are required to developed individualized nutritional and/or medical interventions in patients. 

## Figures and Tables

**Fig. (11):**
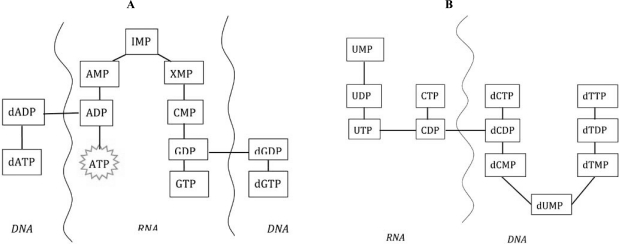
**Abbreviated schematic of metabolic pathways leading to the *de novo* biosynthesis of RNA and DNA precursors.** Purines are synthesized from a branchpoint intermediate, inosine monophosphate (IMP). In the primidine pathway, deoxyuridine and deoxythymidine intermedates are made from deoxycytidine diposphate. ATP, is predominantly synthesized from ADP in the mitochondria, and is the most used cofactor in the cell. Deoxynucleotides are made from ribonucleotides.

**Table 1.** Fragile Sites in the Human Genome

Chr	Locus	Location	R/C	Agent
1	FRA1E	1p21.2	C	Aph
1	FRA1M	1p21.3	R	FolA
1	FRA1D	1p22	C	Aph
1	FRA1L	1p31	C	Aph
1	FRA1C	1p31.2	C	Aph
1	FRA1B	1p32	C	Aph
1	FRA1A	1p36	C	Aph
1	FRA1J	1q12	C	5-Aza
1	FRA1F	1q21	C	Aph
1	FRA1G	1q25.1	C	Aph
1	FRA1K	1q31	C	Aph
1	FRA1H	1q42	C	5-Aza
1	FRA1I	1q44	C	Aph
2	FRA2L	2p11.2	R	FolA
2	FRA2E	2p13	C	Aph
2	FRA2D	2p16.2	C	Aph
2	FRA2C	2p24.2	C	Aph
2	FRA2A	2q11.2	R	FolA
2	FRA2B	2q13	R	FolA
2	FRA2F	2q21.3	C	Aph
2	FRA2K	2q22.3	C	Aph
2	FRA2G	2q31	C	Aph
2	FRA2H	2q32.1	C	Aph
2	FRA2I	2q33	C	Aph
2	FRA2J	2q37.3	C	Aph
3	FRA3B	3p14.2	C	Aph
3	FRA3A	3p24.2	C	Aph
3	FRA3D	3q25	C	Aph
3	FRA3C	3q27	C	Aph
4	FRA4D	4p15	C	Aph
4	FRA4A	4p16.1	C	Aph
4	FRA4B	4q12	C	BrdU
4	FRA4E	4q27	C	Unclas
4	FRA4C	4q31.1	C	Aph
5	FRA5A	5p13	C	BrdU
5	FRA5E	5p14	C	Aph
5	FRA5B	5q15	C	BrdU
5	FRA5D	5q15	C	Aph
5	FRA5F	5q21	C	Aph
5	FRA5C	5q31.1	C	Aph
5	FRA5G	5q35	R	FolA
6	FRA6C	6p22.2	C	Aph
6	FRA6A	6p23	R	FolA
6	FRA6B	6p25.1	C	Aph
6	FRA6D	6q13	C	BrdU
6	FRA6G	6q15	C	Aph
6	FRA6F	6q21	C	Aph
6	FRA6E	6q26	C	Aph
7	FRA7A	7p11.2	R	FolA
7	FRA7D	7p13	C	Aph
7	FRA7C	7p14.2	C	Aph
7	FRA7B	7p22	C	Aph
7	FRA7J	7q11	C	Aph
7	FRA7E	7q21.2	C	Aph
7	FRA7F	7q22	C	Aph
7	FRA7G	7q31.2	C	Aph
7	FRA7H	7q32.3	C	Aph
7	FRA7I	7q36	C	Aph
8	FRA8C	8q24.1	C	Aph
8	FRA8E	8q24.1	R	DistA
8	FRA8F	8q13	R	Unclass
8	FRA8B	8q22.1	C	Aph
8	FRA8A	8q22.3	R	FolA
8	FRA8D	8q24.3	C	Aph
9	FRA9A	9p21	R	FolA
9	FRA9C	9p21	C	BrdU
9	FRA9B	9q32	R	FolA
9	FRA9E	9q32	C	Aph
9	FRA9F	9q12	C	5-Aza
9	FRA9D	9q22.1	C	Aph
10	FRA10B	10q25.2	R	BrdU
10	FRA10E	10q25.2	C	Aph
10	FRA10G	10q11.2	C	Aph
10	FRA10C	10q21	C	BrdU
10	FRA10D	10q22.1	C	Aph
10	FRA10A	10q23.3	R	FolA
10	FRA10F	10q26.1	C	Aph
11	FRA11C	11p15.1	C	Aph
11	FRA11I	11p15.1	R	DistA
11	FRA11E	11p13	C	Aph
11	FRA11D	11p14.2	C	Aph
11	FRA11H	11q13	C	Aph
11	FRA11A	11q13.3	R<R	FolA
11	FRA11F	11q14.2	C	Aph
11	FRA11B	11q23.3	R	FolA
11	FRA11G	11q23.3	C	Aph
12	FRA12A	12q13.1	R	FolA
12	FRA12B	12q21.3	C	Aph
12	FRA12C	12q24	R	BrdU
12	FRA12E	12q24	C	Aph
12	FRA12D	12q24.13	R	FolA
13	FRA13A	13q13.2	C	Aph
13	FRA13B	13q21	C	BrdU
13	FRA13C	13q21.2	C	Aph
13	FRA13D	13q32	C	Aph
14	FRA14B	14q23	C	Aph
14	FRA14C	14q24.1	C	Aph
15	FRA15A	15q22	C	Aph
16	FRA16B	16q22.1	R	DistA
16	FRA16C	16q22.1	C	Aph
16	FRA16E	16p12.1	R	Aph
16	FRA16A	16p13.11	R	FolA
16	FRA16D	16q23.2	C	Aph
17	FRA17A	17p12	R	DistA
17	FRA17B	17q23.1	C	Aph
18	FRA18A	18q12.2	C	Aph
18	FRA18B	18q21.3	C	Aph
19	FRA19B	19p13	R	FolA
19	FRA19A	19q13	C	5-Aza
20	FRA20A	20p11.23	R	FolA
20	FRA20B	20p12.2	C	Aph
22	FRA22B	22q12.2	C	Aph
22	FRA22A	22q13	R	FolA
X	FRAXB	Xp22.31	C	Aph
X	FRAXC	Xq22.1	C	Aph
X	FRAXD	Xq27.1	C	Aph
X	FRAXA	Xq27.3	R	FolA
X	FRAXE	Xq28	R	FolA
X	FRAXF	Xq28		FolA

Chr = chromosome number, R/C= Rare or common, Aph=amphidicolin, Fola= Folic acid, 5-Aza= Azacytidine, Data was compiled from [147, 148] and Genome Database. 1999. Chr = chromosome; R/C = rare/common, Aph = amphidicolin or folic acid, FolA = Folic Acid; 5-AzaC = 5-Azacytidine, BrdU –Bromo-uridine, Unclass = unclassified, DistA = Distamycin( http://ncbi.nlm.nih.gov).

**Table 2.** Summary of Fragile Sites within the Human Genome

Inducer	Common	Rare	Total
Folic	78	22	100
Amphidicolin	78	0	78
BrdU	7	2	9
5-AzaC	4	0	4
Distamycin	0	5	5
Unclassified	1	0	1

**Table 3.** Neurological Diseases Associated with Specific Fragile Sites. Gene Names for Abbreviations are Shown in Table 4

Fragile Site	Associated Gene(s)	Neurological Disease
FRA2A		Mental retardation/schizophrenia
FRA2B		Autism
FRA4F	GRID2	Tremor/Ataxia
FRA6A		Autism
FRA6E	PARK2	Autosomal Juvenile Parkinsonism
FRA6F	LAMA4	Schizophrenia
FRA7I	CNTAP2	Tourette's
FRA9F		Schizophrenia
FRA11B	CBL2	Jacobsen's Syndrome
FRA12A	DIP2B	Autism / Mental retardation
FRA13A	NBEA	Sporadic Autism
FRA15A	RORA	Tremor/Ataxia, Imbalance
FRAXA	FMR1	Fragile X Mental Retardation / FRAXA Tremor Ataxia
FRAXC	IL1RAPL1, DMD	Mental Retardation associated with complex glycerol kinase deficiency
FRAXE	FMR2	Fragile X Mental Retardation (mild)
Global FS Expression	ATR	Seckel syndrome

**Table 4.** Summary of Genes Linked to Schizophrenia and Fragile Sites

GENE				FRAGILE	
NAME	ALIAS	FUNCTION	ADDRESS	SITE	ADDRESS
CHROMOSOME 1					
GSTM1		glutathione S-transferase M1	1p13.3		
GRIK3		glutamate receptor ionotropic	1p34-p33		
**HTR6**		5-hydroxytryptamine (serotonin receptor type 6)	1p36-p35	FRA1A	1p36
**RHD**		Rhesus blood group D antigen	1p36.11	FRA1A	1p36
**MTHFR**		5 10-methylenetetrahydrofolate	1p36.3	FRA1A	1p36
**SCZD9**		schizophrenia disorer 9	1q21-q22	FRA1F	1q21
**SYT11**		Synaptotagamin X1	1q21.2	FRA1F	1q21
**KCNN3**	hSKCa3	potassium intermediate/small c	1q21.3	FRA1F	1q21
RGS4		regulator: g-protein signaling 4	1q23.2		
**IL10**		interleukin 10	1q31-q32	FRA1K	1q31
DISC2		disrupted in schizophrenia 2	1q32.1		
**DISC1**		disrupted in schizophrenia 1	1q42.1	FRA1H	1q42
**CHROMOSOME 2**					
**NOGO**	RTN4	reticulon 4	2p13-p14	FRA2E	2p13
IL1B		interleukin 1 beta	2q14		
**NR4A2**		nuclear receptor subfamily 4, group A, member 2	2q22-23	FRA2K	2q22.3
**CTLA4**		cytotoxic T-lymphocyte-associative protein	2q33	FRA2I	2q33
**CHROMOSOME 3**					
GRM2	GRM2	glutamate receptor metabotropic 2	3p21.31		
CCK		cholecystokinin	3p22-p21.3		
GRM7	GRM7	glutamate receptor metabotropic 7	3p26.1-p25.1		
CHL1 CALL		cell-adhesion molecule with homology to L1CAM	3p26.1		
DRD3		dopamine receptor D3	3q13.3		
**CHROMOSOME 4**					
GABRB1	GABRB1	gamma-aminobutyric acid (GABA) receptor, beta 1	4p12		
**CCKAR**		cholecystokinin A receptor	4p15.1-p15.2	FRA4D	4p15
**DRD5**		dopamine receptor D5	4p16.1	FRA4A	4p16.1
**CHROMOSOME 5**					
**GDNF**		glial cell derived neurotrophic factor	5p13.1-p12	FRA5A	5p13
SCZD1		schizophrenia disorder 1	5q11.2-q13.3		
Homer 1		homer homolog 1 (Drosoph)	5q14.2		
**HTR4**		5-hydroxytryptamine (serotonin) receptor 4	5q31-q33.2	FRA5C	5q31.1
GABRB2		GABA A receptor, beta 2	5q34		
**H2 rec**	HRH2	histamine H2 receptor	5q35.3	FRA5G	5q35
**DRD1**		dopamine receptor D1	5q35.1	FRA5G	5q35
**CHROMOSOME 6**					
**NQO2**		NADPH hydrogenase quinone 2	6pter-q12	FRA6C/A/B	6p22.2/23/25.1
NOTCH4		Notch homolog 4 (Drosophila)	6p21.3		
TNFA		Tumor necrosis factor alpha	6p21.31		
HLA	HLA-A	major histocompatability complex , class I, A	6p21.3		
TNXB		tenascin XB	6p21.3		
DTNBP1		dystrobrevin binding protein 1	6p22.3		
**SCZD3**		schizophrenia disorder 3	6p23	FRA6A	6p23
**SCA1**		spinocerebellar ataxia 1 (oliv)	6p23	FRA6A	6p23
**CB1**	CNR1	Cannabinoid receptor 1	6q14-q15	FRA6G	6q15
**SCZD5**		schizophrenia disorder 5	6q13-q26	FRA6D/E	6q13,q26
**HTR1B**		5-hydroxytryptamine (serotonin) receptor 1B	6q13	FRA6D	6q13
**Fyn kinase**	FYN	FYN oncogene related to SRC, FGR, YES	6q21	FRA6F	6q21
**CHROMOSOME 7**					
**DDC**	DDC	dopa decarboxylase (aromatic L-amino acid decarboxylase)	7p11	FRA7A	7p11.2
NPY		Neuropeptide Y	7p15.1		
**GRM3**		glutamate receptor metabotropi fact. 3	7q21.1-q21.2	FRA7E	7q21.2
**RELN**		reelin	7q22	FRA7F	7q22
**CHROMOSOME 8**					
NRG1		neuregulin 1	8p21-p12		
SCZD6		schizophrenia disorder 6	8p21		
PPP3CC		protein phosphotase 3	8p21.2		
FDZ3		frizzled homolog 3	8p21		
DPYSL2		human dihydroppyrimidinase-related protein 2	8p21-p22		
**CHROMOSOME 9**					
OPRS1	OPRS1	opioid receptor, sigma 1	9p13.2		
DBH		dopamine beta-hydroxylase (dop)	9q34		
GRIN1	NMDA	glutamate receptor ionotropic	9q34.3		
**CHROMOSOME 10**						
**SCA8**		spinocerebellar axia protein 8	10q23.3-24.1	FRA10A	10q23.3
**VMAT2**	SVMT	solute carrier family 18 (vesicular monoamine), member 2	10q25	FRA10B/E	10q25.2
**CHROMOSOME 11**					
**PAX6**		paired box gene 6 (aniridia k)	11p13	FRA11E	11p13
**BDNF**		brain-derived neurotrophic fac	11p13	FRA11E	11p13
**TPH1**		tryptophan hydroxylase	11p15.3-p14	FRA11D	11p14.2
TH		tyrosine hydroxylase	11p15.5		
**cPLA2**	HTATIP2	HIV-1 Tat Interactive Protein 60kDa	11q13	FRA11A/H	11q13.3/ 13
GRIA4		glutamate receptor ionotrophi	11q22		
**DRD2**		Dopamine receptor D2	11q23	FRA11B/G	11q23.3
**HMBS**		hydroxymethylbilane synthase	11q23.3	FRA11B/G	11q23.3
B3GAT		beta-1, 3-Glucronyltransferase-1	11q25		
**CHROMOSOME 12**					
NR2B	GRIN2B	glutamate receptor, ionotropic, N-methyl D-aspartate 2B	12p12		
NTF3	NT3	neurotrophin 3	12p13		
B37	DRPLA	dentatarubral-pallidoluysian atrophy (atrophin- 1)	12p13.31		
**PAH**		phenylalanine hydroxlase	12q22-24.2	FRA12C/E/D	12q24/24.13
**PLA2**		phospholipase A2. group IB	12q23-q24.1	FRA12C/E/D	12q24/24.13
**NOS1**		nitric oxide synthase 1 (neuro)	12q24.2-q24.31	FRA12C/E	12q24
**DAO**	DAOA	d-amino acid oxidase	12q24	FRA12C/E/D	12q24/24.13
**CHROMOSOME 13**					
CAGR1 ***		mab21-like 1 (c. elegans)	13q13	FRA13A	13q13.2
**HTR2**	HTR2/ HTR2a	5-hydorxytryptamine (serotonin) receptor	13q14-q21	FRA13B/C	13q21-q21.2
**SCZD7**		schizophrenia disorder 7	13q32	FRA13D	13q32
G7G72	DAOA	d-amino acid oxidase activator	13q34		
**CHROMOSOME 14**					
NPAS3		neuronal pas domain protein 3	14q12-q13		
CHROMOSOME 15					
HERC2		hect doman and RLD2	15q13		
CHRNA7		cholinergic receptor nicotini	15q14		
SCZD10		schizophrenia disorder 10	15q15		
**CHROMOSOME 16**					
**GRIN2A**		glutamate receptor, ionotropic 2A	16p13.2		
**CHROMOSOME 17**					
SLC6A4	SLC6A4	serotonin transporter	17q11.2-q12		
**ACE**		angiotensin I converting enzym	17q23	FRA17B	17q23.1
**CHROMOSOME 18**					
IMPA2		inositol(myo)-1(or 4)-monophos	18p11.2		
**CHROMOSOME 19**					
SCA6	CACNA1A	calcium channel, voltage dependent, P/Q type, alpha 1A subunit	19p13.2-p13.1	FRA19B	19p13
**APOE**		apolipoprotein E	19q13.2	FRA19A	19q13
**DNMT**		DNA methyltrasnferase 1	19q13.2	FRA19A	19q13
**CHROMOSOME 20**					
**PRNP**		prion protein (p27-30) (Creutz)	20pter-p12	FRA20B	20p12.2
**SNAP-25**		synaptosomal-associated protein 25kDa	20p12-p11.2	FRA20B/A	20p12.2/11.23
**CHGB**		chromogranin B ( secretogranin 1)	20pter-p12	FRA20B	20p12.2
**CHROMOSOME 22**						
COMT		catechol-O-methyltransferase	22q11.21		
SNAP29		synaptosomal-associated protein	22q11.21		
PCQAP		PC2 (positive cofactor 2 mult	22q11.2		
PRODH/DGCR6		DiGeorge Syndrome critical region, gene 6	22q11.21		
UFD1L		ubiquitin fusion degradation 1	22q11.21		
ZNF74		zinc finger protein 74 (Cos52)	22q11.21		
**APOL-4**		apolipoprotien L-4	22q11.2-13.2	FRA22A/B	22q12.2/13
**APOL-2**		apolipoprotien L2	22q12	FRA22B	22q12.2
SYN3		synaptin 3	22q12.3		
TIMP3		tissue inhibitor of metalloprot.3	22q12.3		
YWHAH		tyrosine 3-monooxygenase/trypt	22q12.3		
**APOL-1**		apolipoprotein L1	22q13.1	FRA22A	22q13
**SYNGR1**		synaptogyrin 1	22q13.1	FRA22A	22q13
**CYP2D6**		cytochrome P450 family 2 sub	22q13.1	FRA22A	22q13
**IL2RB**		interleukin 2 receptor beta	22q13/13.1	FRA22A	2222q13
**BZRP**	BZRP	benzodiazapine receptor (peripheral)	22q13.31	FRA22A	22q13
**WKL1**	MLC1	megalencephalic leukoencephalopathy with subcortical cysts 1	22q13.33	FRA22A	22q13
**X CHROMOSOME**					
HTR2C		5-hydorxytryptamine (serotonin) receptor 2C	Xq24		
**L1CAM**		L1 cell adhesion molecule	Xq28	FRAXE/F	Xq28

Studies were obtained from the National Institute of Health’s database linking specific genes to schizophrenia at http://www.geneticassociationdb.com. In addition, a Pubmed search using the keywords "gene AND schizophrenia" yielded more unique studies. The genes found using these two methods were then searched more exclusively using the keywords “ gene name” AND schizophrenia” in order to more thoroughly assess whether at least one positive association was found between a gene and schizophrenia. Genes are organized by chromosomal locations, and appear in bold when co-localizing with a chromosomal fragile sites. The co-localizing fragile site name and address is shown. More information can be found at http://schizogad.bu.edu.

**Table 5.** Odds Ratio of Genetics and Environmental Factors Linked to Schizophrenia. Adapted from [149]

Factor		Odds Ratio
Place/time of birth	Winter	1.2
	Urban	1.5
Infection	Influenza	2.0
	Respiratory	2.2
	Rubella	5.2
	Poliovirus	1.1
	CNS	4.0
Prenatal	Famine	2.0
	Bereavement	6.2
	Flood	1.8
	Unwantedness	2.4
	Maternal depr	1.8
Obstetric	Rh incompatibility	2.8
	Hypoxia	3.0
	CNS damage	7.0
	Low birth weight	1.6
	Pre-eclampsia	2.5
Genetics	Family history	9.7
